# The relative deficit of GDF15 in adolescent girls with PCOS can be changed into an abundance that reduces liver fat

**DOI:** 10.1038/s41598-021-86317-9

**Published:** 2021-03-29

**Authors:** Francis de Zegher, Marta Díaz, Joan Villarroya, Montserrat Cairó, Abel López-Bermejo, Francesc Villarroya, Lourdes Ibáñez

**Affiliations:** 1 Department of Development and Regeneration, University of Leuven, 3000 Leuven, Belgium; 2 Endocrinology Department, Institut de Recerca Pediàtric, Hospital Sant Joan de Déu, University of Barcelona, Passeig de Sant Joan de Déu, 2, Esplugues, 08950 Barcelona, Spain; 3 Centro de Investigación Biomédica en Red de Diabetes y Enfermedades Metabólicas Asociadas (CIBERDEM), ISCIII, Madrid, Spain; 4 Department of Biochemistry and Molecular Biomedicine, University of Barcelona, Barcelona, Spain; 5 Centro de Investigación Biomédica en Red de Fisiopatología de la Obesidad y Nutrición (CIBERObn), ISCIII, Madrid, Spain; 6 Pediatric Endocrinology Research Group, Girona Institute for Biomedical Research (IDIBGI), Dr. Josep Trueta Hospital, 17007 Girona, Spain

**Keywords:** Biomarkers, Endocrinology

## Abstract

A prime concern of young patients with Polycystic Ovary Syndrome (PCOS) is the control of body adiposity, given their tendency to gain weight and/or their difficulty to lose weight. Circulating growth-and-differentiation factor-15 (GDF15) facilitates the control of body weight via receptors in the brainstem. C-reactive protein (CRP) and insulin are endogenous GDF15 secretagogues. We hypothesised that PCOS in non-obese adolescents is characterised by low concentrations of circulating GDF15, when judged by the degree of CRP and insulin drive. GDF15 was added as a post-hoc endpoint of two previously reported, randomised studies in non-obese adolescent girls with PCOS (N = 58; 60% normal weight; 40% overweight) who received either an oral oestroprogestogen contraceptive (OC), or a low-dose combination of spironolactone-pioglitazone-metformin (SPIOMET) for 1 year; subsequently, all girls remained untreated for 1 year. Adolescent girls with regular menses (N = 20) served as healthy controls. Circulating GDF15, CRP and fasting insulin were assessed prior to treatment, and halfway the on- and post-treatment years. Pre-treatment, the absolute GDF15 concentrations were normal in PCOS girls, but their relative levels were markedly low, in view of the augmented CRP and insulin drives. OC treatment was accompanied by a near-doubling of circulating GDF15 (on average, from 296 to 507 pg/mL) and CRP, so that the relative GDF15 levels remained low. SPIOMET treatment was accompanied by a 3.4-fold rise of circulating GDF15 (on average, from 308 to 1045 pg/mL) and by a concomitant lowering of CRP and insulin concentrations towards normal, so that the relative GDF15 levels became markedly abundant. Post-OC, the relatively low GDF15 levels persisted; post-SPIOMET, the circulating concentrations of GDF15, CRP and insulin were all normal. BMI remained stable in both treatment groups. Only SPIOMET was accompanied by a reduction of hepato-visceral fat (by MRI) towards normal. In conclusion, early PCOS was found to be characterised by a relative GDF15 deficit that may partly explain the difficulties that young patients experience to control their body adiposity. This relative GDF15 deficit persisted during and after OC treatment. In contrast, SPIOMET treatment was accompanied by an absolute and a relative abundance of GDF15, and followed by normal GDF15, CRP and insulin concentrations. The present findings strengthen the rationale to raise the concentrations of circulating GDF15 in early PCOS, for example with a SPIOMET-like intervention that attenuates low-grade inflammation, insulin resistance and ectopic adiposity, without necessarily lowering body weight.

**Clinical trial registries**: ISRCTN29234515 and ISRCTN11062950.

## Introduction

The key features of adolescent Polycystic Ovary Syndrome (PCOS) are androgen excess (clinical and biochemical) and menstrual irregularity (oligo-amenorrhea) > 2 years beyond menarche^1^. However, the top-ranked concern of young PCOS patients is the control of body adiposity, given their tendency to gain weight and/or their difficulty to lose weight^2^.

Circulating Growth-and-Differentiation Factor 15 (GDF15) can reduce appetite^3^ via specific receptors in the area postrema and nucleus tractus solitarius of the brainstem^4^. In the absence of acute illness, C-reactive protein (CRP) and insulin are among the endogenous GDF15 secretagogues, respectively in endothelial cells^5^ and in the liver^6^. Metformin is an exogenous GDF15 secretagogue^7–9^, whose principal site of GDF15-releasing action is currently thought to be in the gut, in particular in the colon^10^.

We tested the hypothesis that PCOS in non-obese adolescents is characterised by low concentrations of circulating GDF15, when judged by the degree of CRP and insulin drive. In addition, we took advantage of a randomised clinical trial in non-obese adolescent girls with PCOS to study the effects of an oral oestroprogestogen contraceptive (OC) on circulating GDF15, and also the effects of a low-dose combination of spironolactone-pioglitazone-metformin (SPIOMET) that reduces ectopic adiposity, attenuates the androgen excess, and raises the ovulation rate, without necessarily changing body weight^11,12^. OC and SPIOMET treatment are known to have opposing effects on circulating insulin and CRP^12,13^.

## Methods

GDF15 was added as a post-hoc endpoint of two randomised studies in adolescent girls with PCOS; these open-label pilot studies (ISRCTN29234515; http://www.controlled-trials.com/ISRCTN29234515; and ISRCTN11062950; http://www.isrctn.com/ISRCTN11062950), compared the on- and post-treatment effects of OC *vs* SPIOMET; pooled results have been reported in detail, not only of the primary endpoint (post-treatment ovulation rate) but also of secondary endpoints such as hirsutism score, androgens, carotid intima-media thickness, and body composition, including hepatic and visceral fat^12–14^.

In brief, both studies were conducted in the Endocrinology Unit of Sant Joan de Déu University Hospital, Barcelona, Spain. Recruitment was biased against overweight/obesity since, in this setting, overweight/obese adolescents are mainly referred to the Obesity Unit^12^. In each study, an on-treatment year was followed by a post-treatment year; due to the limited availability of spare serum, we focus here on results prior to treatment, halfway the on-treatment year, and halfway the post-treatment year. At these timepoints, serum was available for GDF15 measurement in 94% (58/62) of the study-completing patients, which corresponds to 82% (58/71) of the enrolled patients (Suppl Fig. 1, flow chart in ESM).

Age-matched, healthy girls (n = 20) recruited in nearby schools for the original studies (11,12) served as controls; all had regular menses and a gynaecological age > 2.0 years; none was hirsute, had a chronic disease, or took medication.

Inclusion criteria were hirsutism (modified Ferriman-Gallwey score > 8), oligomenorrhea (menstrual intervals > 45 day), gynaecological age > 2.0 years, and absence of sexual activity. Exclusion criteria were 21‐hydroxylase deficiency; glucose intolerance or diabetes; evidence of thyroid, liver, or kidney dysfunction; hyperprolactinemia; and any prior use of medications affecting gonadal/adrenal function, or carbohydrate/lipid metabolism.

Mediterranean diet and regular exercise were recommended to all study participants; OC treatment consisted of 20 μg ethinyloestradiol plus 100 mg levonorgestrel (21/28 days), and placebo (7/28 days); SPIOMET treatment consisted of spironolactone 50 mg (sufficient to raise the activity of brown adipose tissue^15^), pioglitazone 7.5 mg, and metformin 850 mg, taken together, once daily at dinner time.

Birthweight was retrieved from medical records. Blood sampling was performed in the early morning, after an overnight fast, in the follicular phase of the menstrual cycle (day 3–7) or after two months of amenorrhea. GDF15 was measured in duplicate by ELISA (R&D Systems, Minneapolis, USA) with intra- and inter-assay coefficients of variation (CVs) below 6%^16^. CRP was measured with a highly sensitive method (Architect c8000; Abbott, Wiesbaden, Germany), and insulin by immunochemiluminiscence (Immulite 2000, Diagnostic Products, Los Angeles, USA). HOMA-insulin resistance (HOMA-IR) was calculated as [fasting insulin in mU/L] x [fasting glucose in mg/dL]/405. High-molecular-weight (HMW) adiponectin was measured by ELISA (Linco, St. Louis, MO). Abdominal fat (subcutaneous and visceral) and hepatic fat were assessed by magnetic resonance imaging (MRI) using a multiple‐slice MRI 1.5 T scan (Signa LX Echo Speed Plus Excite, General Electric, Milwaukee, Wisconsin), as described^11^.

Statistical analyses were performed with SPSS 23.0 (IBM, Armonk, New York, USA). All the analysed variables passed the Kolmogorov–Smirnov test for normality of distribution. Comparisons within and between groups at each timepoint were performed using paired and unpaired *t*-tests, respectively. P < 0.05 was considered significant. Data are presented as mean ± SEM.

The studies were conducted after approval by the Institutional Review Board of Sant Joan de Déu Hospital, after written informed consent by parents, and after assent by each participating girl.

All methods were carried out in accordance with relevant guidelines and regulations.

## Results

**Figure 1:**
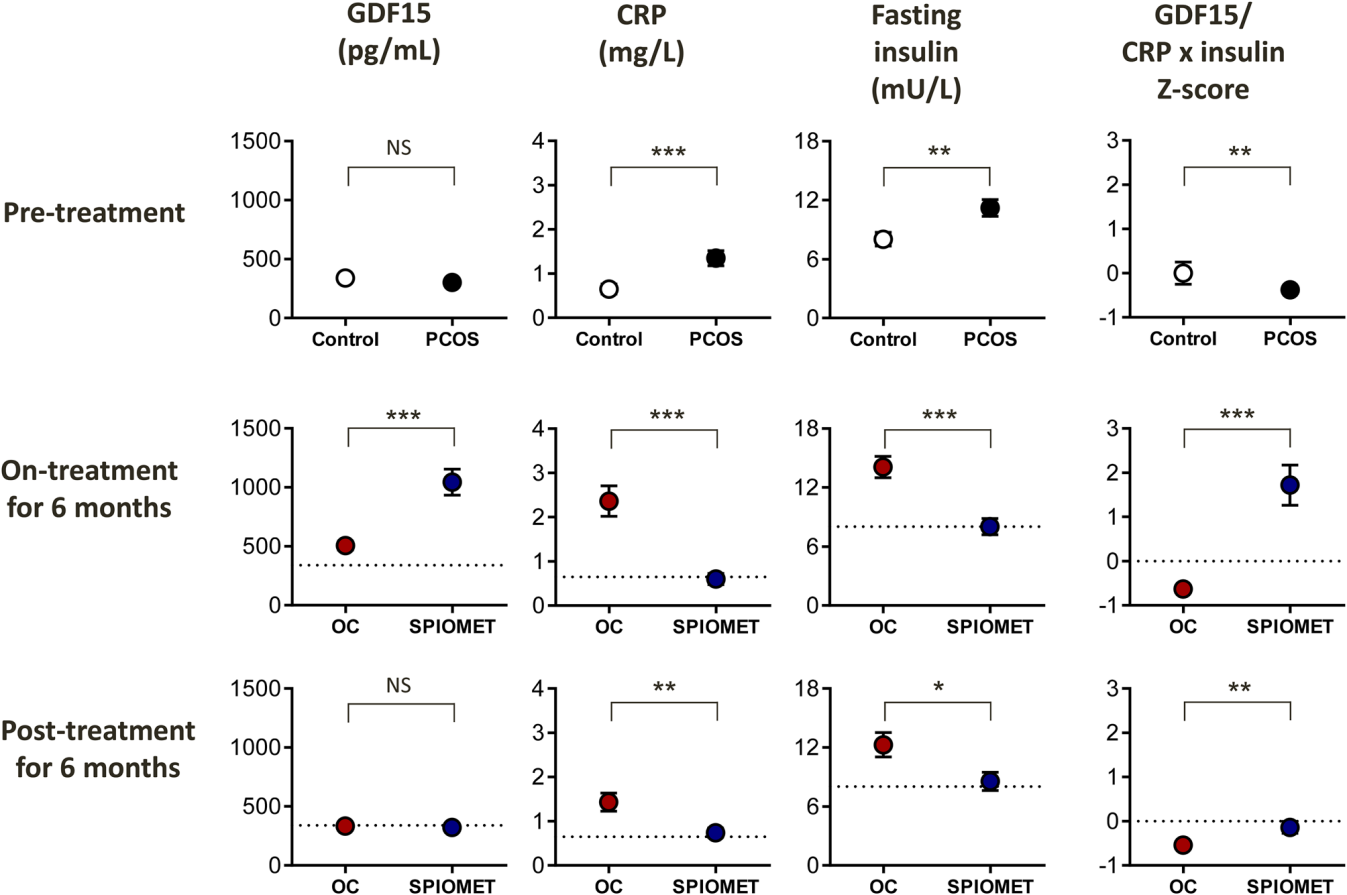
Circulating levels of GDF15, C-reactive protein (CRP) and fasting insulin are shown, as well as the Z-score of the ratio of GDF15 to the product of CRP and fasting insulin. Results are expressed as mean ± SEM. Upper panels show the pre-treatment results in healthy controls (N = 20) and in adolescent girls with polycystic ovary syndrome (PCOS, N = 58). Middle panels show the results in girls with PCOS, 6 months after starting a randomised treatment with either an oral contraceptive containing ethinyloestradiol plus levonorgestrel (OC; red circles N = 29) or a low-dose combination of spironolactone, pioglitazone, and metformin (SPIOMET; blue circles, N = 29) for 12 months. Lower panels show the results in girls with PCOS, 6 months after stopping OC or SPIOMET treatment. * p < 0.05, **p < 0.01, ***p < 0.001 between control and PCOS (upper panels), or between OC and SPIOMET (middle and lower panels).

The results are summarised in Table 1 and depicted in Fig. 1.

**Table 1:** Selected data from adolescent girls with polycystic ovary syndrome (PCOS) who were randomized to receive either an oral contraceptive consisting of ethinylestradiol-levonorgestrel (N=29) or a low-dose combination of spironolactone-pioglitazone-metformin (SPIOMET, N=29) for 12 months (12 mo), and who were subsequently followed without treatment for 12 months. Longitudinal results of randomised subgroups are shown: pre-treatment (0 mo), on-treatment for 6 months (6 mo), and post-treatment for 6 months (18 mo).

	Controls (N = 20)	All PCOS (N = 58)	Ethinylestradiol-levonorgestrel On-treatment for 12 mo, post-treatment for 12 mo					SPIOMET On-treatment for 12 mo, post-treatment for 12 mo				
			Pre-treatment ^a^	On-treatment for 6 months	Post-treatment for 6 months	Δ 0–6 mo	Δ 6–18 mo	Pre-treatment ^a^	On-treatment for 6 months	Post-treatment for 6 months	Δ 0–6 mo	Δ 6–18 mo
Birthweight Z-score	0.1 ± 0.3	−0.6 ± 0.1**	−0.7 ± 0.3	–	–	–	–	−0.6 ± 0.2	–	–	–	–
Age at Menarche (year)	12.2 ± 0.2	11.6 ± 0.1**	11.6 ± 0.1	–	–	–	–	11.6 ± 0.2	–	–	–	–
Age (year)	16.0 ± 0.3	15.7 ± 0.2	15.8 ± 0.3	–	–	–	–	15.6 ± 0.3	–	–	–	–
BMI Z-score^¶^	0.0 ± 0.2	0.8 ± 0.1**	0.9 ± 0.2	1.0 ± 0.2	1.1 ± 0.3	0.1 ± 0.1	0.1 ± 0.1	0.7 ± 0.2	0.7 ± 0.2	0.8 ± 0.2	0.0 ± 0.1	0.1 ± 0.1
Δ Z-score Birthweight to BMI	−0.1 ± 0.3	1.4 ± 0.2***	1.6 ± 0.3	1.7 ± 0.3	1.8 ± 0.3	0.1 ± 0.1	0.1 ± 0.1	1.3 ± 0.3	1.3 ± 0.3	1.4 ± 0.3	0.0 ± 0.1	0.1 ± 0.1
GDF15 (pg/mL)	339 ± 20	302 ± 17	296 ± 29	507 ± 37^d^	334 ± 22^d^	211 ± 45	−173 ± 38	308 ± 16	1045 ± 111^d^	321 ± 13^d^	737 ± 121^ g^	−724 ± 105^ g^
Z-score GDF15 (pg/mL)	0.0 ± 0.2	−0.4 ± 0.2	−0.5 ± 0.3	1.8 ± 0.4^d^	−0.1 ± 0.2^d^	2.3 ± 0.5	−1.9 ± 0.4	−0.3 ± 0.2	7.7 ± 1.2^d^	−0.2 ± 0.1^d^	8.0 ± 0.3^ g^	−7.9 ± 1.1^ g^
CRP (mg/L)	0.7 ± 0.1	1.3 ± 0.2***	1.3 ± 0.2	2.4 ± 0.3^c^	1.4 ± 0.2^c^	1.1 ± 0.3	−1.0 ± 0.3	1.4 ± 0.3	0.6 ± 0.1^c^	0.7 ± 0.1	−0.8 ± 0.3^ g^	0.1 ± 0.1^f^
Fasting Insulin (pmol/L)	8.0 ± 0.7	11.2 ± 0.8**	12.4 ± 1.3	14.1 ± 1.1^b^	12.3 ± 1.2^b^	1.7 ± 0.9	−1.8 ± 0.8	10.0 ± 1.0	8.0 ± 0.8^b^	8.6 ± 0.9	−2.0 ± 0.7^f^	0.6 ± 0.9^e^
Z-score ratio GDF15/CRP x insulin	0.0 ± 0.3	−0.5 ± 0.1**	−0.5 ± 0.1	−0.6 ± 0.0	−0.5 ± 0.1	−0.1 ± 0.1	0.1 ± 0.0	−0.5 ± 0.1	1.7 ± 0.5^d^	−0.1 ± 0.1^d^	2.2 ± 0.5^ g^	−1.8 ± 0.4^ g^
Total testosterone (nmol/L)	0.5 ± 0.2	1.4 ± 0.1*	1.3 ± 0.1	0.7 ± 0.1^b^	1.5 ± 0.2^c^	−0.6 ± 0.1	0.8 ± 0.2	1.5 ± 0.2	0.9 ± 0.1^b^	1.1 ± 0.1	−0.6 ± 0.1	0.2 ± 0.1^f^
Free androgen index^#^	1.1 ± 0.3	5.2 ± 0.5***	4.7 ± 0.5	1.4 ± 0.2^c^	5.1 ± 1.0^c^	−3.3 ± 0.1	3.7 ± 1.2	5.5 ± 0.8	2.9 ± 0.3^c^	2.9 ± 0.4	−2.6 ± 0.1	0.0 ± 0.3^ g^
HDL-cholesterol (mmol/L)	1.4 ± 0.1	1.3 ± 0.1	1.3 ± 0.1	1.3 ± 0.1	1.4 ± 0.1^b^	0.0 ± 0.1	0.1 ± 0.1	1.3 ± 0.1	1.4 ± 0.1^c^	1.3 ± 0.1	0.1 ± 0.1	−0.1 ± 0.1^f^
LDL-cholesterol (mmol/L)	2.2 ± 0.1	2.3 ± 0.1	2.3 ± 0.1	2.6 ± 0.1^d^	2.4 ± 0.1^c^	0.3 ± 0.1	−0.2 ± 0.1	2.3 ± 0.1	2.2 ± 0.1	2.1 ± 0.1	−0.1 ± 0.1^f^	−0.1 ± 0.1
Triglycerides (mmol/L)	0.6 ± 0.1	0.7 ± 0.1*	0.6 ± 0.1	0.7 ± 0.1	0.6 ± 0.1^c^	0.1 ± 0.1	−0.1 ± 0.1	0.7 ± 0.1	0.7 ± 0.1	0.7 ± 0.1	0.0 ± 0.1^e^	0.0 ± 0.1^f^.
HMW adiponectin (mg/L)	8.1 ± 1.0	7.1 ± 0.6	6.7 ± 0.6	8.9 ± 1.3	7.6 ± 0.9	2.2 ± 1.2	−1.3 ± 1.0	7.0 ± 0.9	14.1 ± 1.8^b^	15.7 ± 2.9	7.1 ± 2.1^e^	1.6 ± 3.2
HOMA-IR	1.7 ± 0.2	2.4 ± 0.2*	2.7 ± 0.3	3.1 ± 0.3^b^	2.8 ± 0.3	0.4 ± 0.2	−0.3 ± 0.3	2.1 ± 0.2	1.7 ± 0.2^b^	1.8 ± 0.2	−0.4 ± 0.2^f^	0.1 ± 0.2
Abd MRI subcutaneous fat (cm^2^)	103 ± 13	168 ± 13***	172 ± 19	173 ± 19	180 ± 21	1 ± 6	7 ± 9	164 ± 18	163 ± 19	168 ± 22	−1 ± 6	5 ± 9
Visceral fat (cm^2^)	27 ± 2	43 ± 2***	42 ± 3	44 ± 4	40 ± 3	2 ± 2	−4 ± 3	44 ± 4	35 ± 2^c^	36 ± 2	−9 ± 3^f^	1 ± 2
Liver fat (%)	10 ± 1	17 ± 1***	17 ± 1	19 ± 1^b^	17 ± 1^b^	2 ± 1	−2 ± 2	18 ± 1	12 ± 1^d^	9 ± 1^c^	−6 ± 1^ g^	−3 ± 1

Values are mean ± SEM.

*BMI* body mass index, *GDF15* growth differentiation factor 15, *CRP* C-reactive protein, *HDL* high-density lipoprotein, *LDL-C* low-density lipoprotein, *HMW adiponectin* high-molecular-weight adiponectin, *HOMA-IR* homeostasis model assessment-insulin resistance, *Abd MRI* abdominal magnetic resonance imaging.

^#^Testosterone (nmol/L) /SHBG (nmol/L).

^¶^Among the 58 girls with PCOS, 23 (40%) were overweight (BMI Z-score between 1.0 and 2.0).

^a^No significant differences between randomised pre-treatment subgroups.

^b^p <0.05, ^c^p≤0.01 and ^d^p ≤0.001 within subgroups for 0–6 months & 6–18 months change (Δ).

^e^p <0.05, ^f^p ≤0.01, ^g^p ≤0.001 between subgroups for 0–6 months & 6–18 change (Δ).

^*^ p <0.05, ^**^p≤0.01, ^***^p ≤0.001 between controls and PCOS.

### Control versus PCOS

Non-obese adolescents with PCOS experienced a mismatch between prenatal and postnatal weight gain, were younger at menarche, and presented an abdominal excess of fat in subcutaneous, visceral and hepatic depots; they had low-normal concentrations of circulating GDF15, despite markedly raised levels of CRP and insulin, suggestive of a relative GDF15 deficit.

### OC versus SPIOMET

OC and SPIOMET treatment were accompanied, respectively, by 1.7- and 3.4-fold rises of circulating GDF15. The difference between these increments is even more striking when the divergent courses of circulating CRP and insulin are taken into account: on OC, the concentrations of these two GDF15 secretagogues increased further above normal whereas, on SPIOMET, they decreased towards normal.

Post-OC, the circulating concentrations of GDF15, CRP and insulin returned towards baseline levels; post-SPIOMET, there appeared to be prolonged benefits: GDF15 returned also to baseline levels but CRP, insulin and liver fat remained normal, while circulating HMW adiponectin remained elevated.

## Discussion

Recent evidence establishing GDF15 as a mediator of metformin’s effects on body weight and energy balance^7–9^, prompted a post-hoc analysis of circulating GDF15 concentrations in adolescent girls with PCOS before, during and after randomised treatments either with a standard intervention silencing the gonadotropic axis (OC), or with a low-dose combination of generic medications targeting a reduction of ectopic fat (SPIOMET)^12–14^.

Pre-treatment concentrations of circulating GDF15 were found to be similar in healthy controls and in adolescents with PCOS. However, the presence of two natural GDF15 secretagogues (CRP and insulin) was much higher in the circulation of adolescents with PCOS. After accounting for this marked difference, the GDF15 levels in PCOS could be interpreted as being relatively low for the endogenous drive, in other words, as pointing towards a relative GDF15 deficit in non-obese PCOS. It remains to be explored to which extent this relative GDF15 deficit explains why young PCOS patients tend to gain weight and to experience difficulties to lose weight.

On-treatment concentrations of circulating GDF15 increased 1.7-fold with OC and 3.4-fold with SPIOMET intervention. However, the two treatment groups changed not only their CRP but also their insulin concentrations in opposing directions, so that OC intake resulted in a maintenance of the relative GDF15 deficit, whereas SPIOMET intake was associated with a striking reversal into a state of absolute and relative GDF15 abundance, and with a concomitant loss of liver fat^12–14^.

Endogenously rising concentrations of circulating GDF15, CRP and fasting insulin are each among the top-ranked biomarkers of aging and age-related disease^17^. OC treatment in adolescent girls with PCOS was found to raise the circulating concentrations of each of these three biomarkers. It remains to be studied whether such collective increments, when prolonged through the early stages of PCOS, do indeed imply an acceleration of aging processes, potentially with long-term sequelae.

The normal range of circulating GDF15 is generally considered to be 0.2–1.2 ng/mL^18^, with higher levels in early life^16^ and also in later life^17^. Thus, SPIOMET increased the GDF15 levels in adolescents with PCOS from low-normal (0.2–0.4 ng/mL) into high-normal range (0.8–1.2 ng/mL); it reduced liver steatosis, insulin resistance and low-grade inflammation, and raised adiponectinaemia, without causing anorexia or a loss of body weight. Hence, the range wherein circulating GDF15 can exert steatosis-reducing actions and other metabolic benefits^19^ in adolescents may be much lower (0.8–1.2 ng/mL) than the range wherein it causes anorexia and weight loss^9,18^. Therefore, the age range with the highest potential for benefit from metformin or SPIOMET-like interventions may be between childhood^16,20^ and early adulthood.

Post-treatment concentrations of circulating GDF15 were comparable to the normal pre-treatment levels, in both treatment groups. Post-OC, the CRP and insulin concentrations returned to the high pre-treatment levels, so that the relative GDF15 deficit did persist. Post-SPIOMET, the CRP and insulin concentrations were both normal, so that the relative GDF15 deficit was no longer observed. It remains to be further clarified how a transient SPIOMET intervention in non-obese adolescents with PCOS can elicit endocrine, metabolic and body-composition normalisations that extend beyond the time window of active treatment. Candidate mechanisms include prolonged changes in the profiles of brown adipokines^15^ and microRNAs^21^.

One limitation of the present study is the relatively low number of healthy, age-matched controls who allowed to establish a range of circulating GDF15 concentrations indicative of normality. Both the average (0.3 ng/mL) and the range (approximately 0.2–0.4 ng/mL) of these indicative values compared to those observed in young, normal-weight or overweight women^22^. Another limitation is that the design of the randomised study does not allow to infer whether the increment of circulating GDF15 concentrations is in OC-treated adolescents mainly due to the oestrogen or to the progestogen, and is in SPIOMET-treated adolescents mainly due to spironolactone, to pioglitazone, or to metformin.

In conclusion, a relative deficit of circulating GDF15 may contribute to explain the difficulties that young PCOS patients experience in the control of their body adiposity. OC treatment is accompanied by increments of circulating GDF15, CRP and fasting insulin: this is a profile of accelerated aging^20^. SPIOMET treatment is accompanied by an absolute and relative abundance of GDF15, and is followed by normal GDF15, CRP and insulin concentrations. The present findings strengthen the rationale to raise the concentrations of circulating GDF15 in early PCOS, for example by a SPIOMET-like intervention that attenuates ectopic adiposity, insulin resistance and low-grade inflammation, without necessarily lowering body weight.

## Supplementary Information

Supplementary Figure 1.

## Data Availability

The datasets generated during and/or analysed during the current study are available from the corresponding author on reasonable request.

## Abbreviations

CRP: C-reactive protein
GDF15: Growth-and-differentiation factor-15
HMW: High molecular weight
MRI: Magnetic resonance imaging
OC: Oral oestroprogestogen contraceptive
PCOS: Polycystic ovary syndrome
SPIOMET: Combo of spironolactone (50 mg/day), pioglitazone (7.5 mg/day), metformin (850 mg/day)
