# Author Correction: The relative deficit of GDF15 in adolescent girls with PCOS can be changed into an abundance that reduces liver fat

**DOI:** 10.1038/s41598-021-99918-1

**Published:** 2021-10-08

**Authors:** Francis de Zegher, Marta Díaz, Joan Villarroya, Montserrat Cairó, Abel López‑Bermejo, Francesc Villarroya, Lourdes Ibáñez

**Affiliations:** 1 Department of Development and Regeneration, University of Leuven, 3000 Leuven, Belgium; 2 Endocrinology Department, Institut de Recerca Pediàtric, Hospital Sant Joan de Déu, University of Barcelona, Passeig de Sant Joan de Déu, 2, Esplugues, 08950 Barcelona, Spain; 3 Centro de Investigación Biomédica en Red de Diabetes y Enfermedades Metabólicas Asociadas (CIBERDEM), ISCIII, Madrid, Spain; 4 Department of Biochemistry and Molecular Biomedicine, University of Barcelona, Barcelona, Spain; 5 Centro de Investigación Biomédica en Red de Fisiopatología de la Obesidad y Nutrición (CIBERObn), ISCIII, Madrid, Spain; 6 Pediatric Endocrinology Research Group, Girona Institute for Biomedical Research (IDIBGI), Dr. Josep Trueta Hospital, 17007 Girona, Spain

Correction to: *Scientific Reports* 10.1038/s41598-021-86317-9, published online 29 March 2021

The Funding section in the original version of this Article was omitted. The Funding section now reads:

“This work was supported by a grant PERIS-SLT006/17/00140 from the AQU, Generalitat de Catalunya, Spain”.

The original Article has been corrected.

