# Supplementary material for: The relative deficit of GDF15 in adolescent girls with PCOS can be changed into an abundance that reduces liver fat
Source: Sci Rep. 2021 Mar 29;11:7018. doi: 10.1038/s41598-021-86317-9 (PMC8007831; doi:10.1038/s41598-021-86317-9)
Supplement: Supplementary file 1 — Supplementary Figure 1. [file 41598_2021_86317_MOESM1_ESM.pptx]

## Slide 1
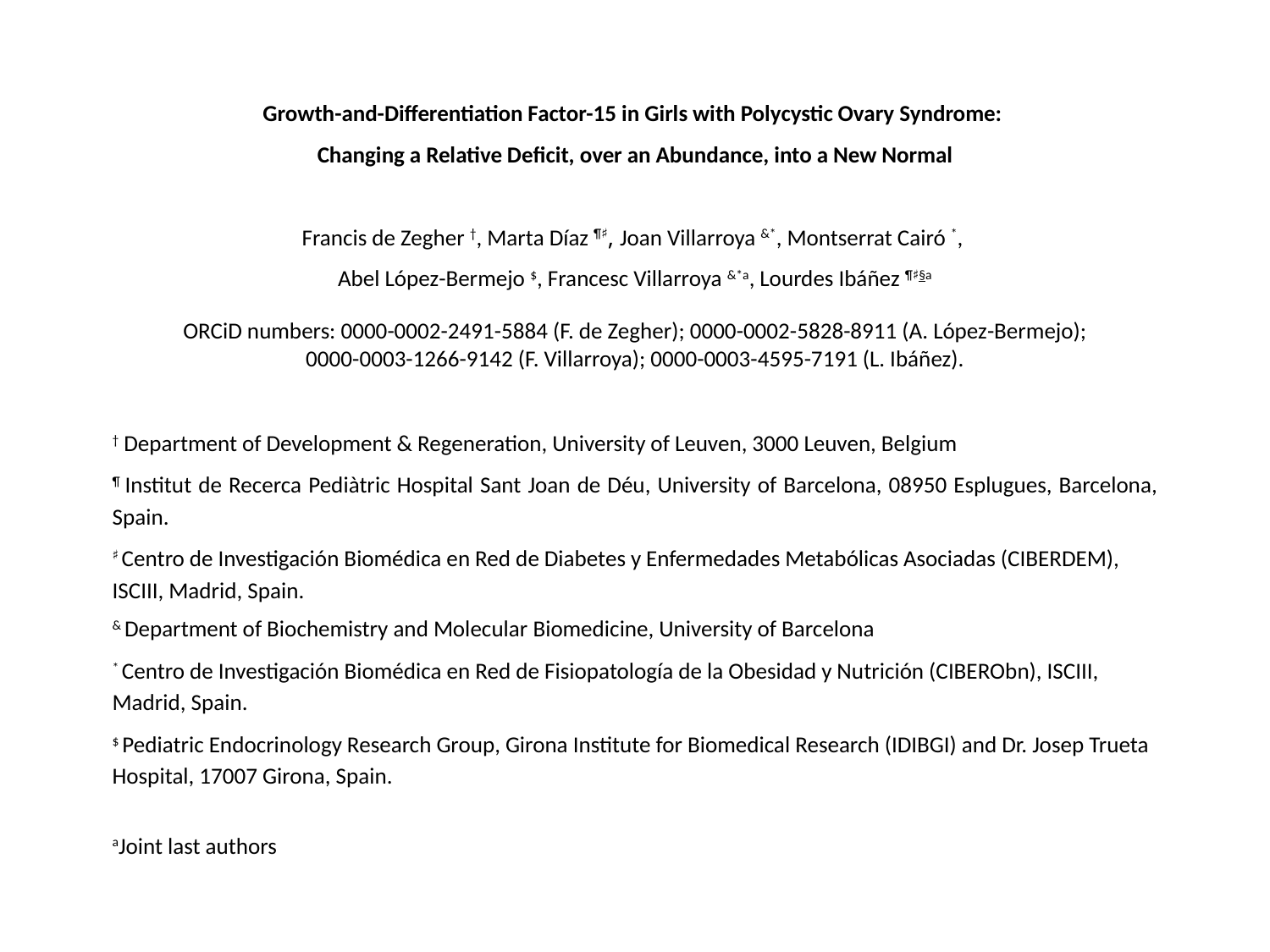

Growth-and-Differentiation Factor-15 in Girls with Polycystic Ovary Syndrome:
Changing a Relative Deficit, over an Abundance, into a New Normal
Francis de Zegher †, Marta Díaz ¶♯, Joan Villarroya &*, Montserrat Cairó *,
Abel López-Bermejo $, Francesc Villarroya &*a, Lourdes Ibáñez ¶♯§a
ORCiD numbers: 0000-0002-2491-5884 (F. de Zegher); 0000-0002-5828-8911 (A. López-Bermejo);
0000-0003-1266-9142 (F. Villarroya); 0000-0003-4595-7191 (L. Ibáñez).
† Department of Development & Regeneration, University of Leuven, 3000 Leuven, Belgium
¶ Institut de Recerca Pediàtric Hospital Sant Joan de Déu, University of Barcelona, 08950 Esplugues, Barcelona, Spain.
♯ Centro de Investigación Biomédica en Red de Diabetes y Enfermedades Metabólicas Asociadas (CIBERDEM), ISCIII, Madrid, Spain.
& Department of Biochemistry and Molecular Biomedicine, University of Barcelona
* Centro de Investigación Biomédica en Red de Fisiopatología de la Obesidad y Nutrición (CIBERObn), ISCIII, Madrid, Spain.
$ Pediatric Endocrinology Research Group, Girona Institute for Biomedical Research (IDIBGI) and Dr. Josep Trueta Hospital, 17007 Girona, Spain.
aJoint last authors

## Slide 2
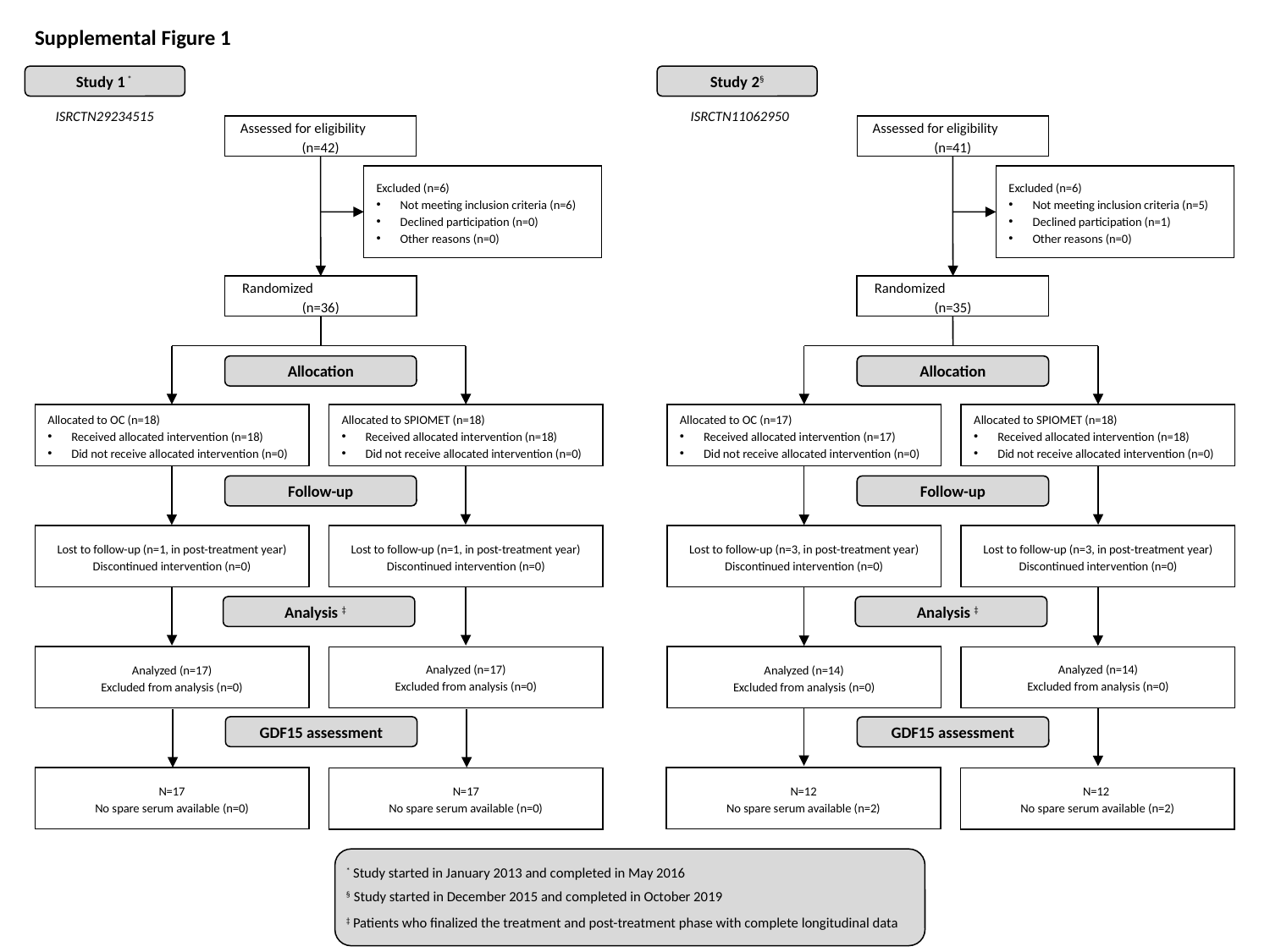

Supplemental Figure 1
Study 1 *
Study 2§
ISRCTN29234515
ISRCTN11062950
Assessed for eligibility (n=42)
Excluded (n=6)
Not meeting inclusion criteria (n=6)
Declined participation (n=0)
Other reasons (n=0)
Randomized (n=36)
Allocation
Allocated to OC (n=18)
Received allocated intervention (n=18)
Did not receive allocated intervention (n=0)
Allocated to SPIOMET (n=18)
Received allocated intervention (n=18)
Did not receive allocated intervention (n=0)
Follow-up
Lost to follow-up (n=1, in post-treatment year)
Discontinued intervention (n=0)
Lost to follow-up (n=1, in post-treatment year)
Discontinued intervention (n=0)
Analysis ‡
Analyzed (n=17)
Excluded from analysis (n=0)
Analyzed (n=17)
Excluded from analysis (n=0)
Assessed for eligibility (n=41)
Excluded (n=6)
Not meeting inclusion criteria (n=5)
Declined participation (n=1)
Other reasons (n=0)
Randomized (n=35)
Allocation
Allocated to OC (n=17)
Received allocated intervention (n=17)
Did not receive allocated intervention (n=0)
Allocated to SPIOMET (n=18)
Received allocated intervention (n=18)
Did not receive allocated intervention (n=0)
Follow-up
Lost to follow-up (n=3, in post-treatment year)
Discontinued intervention (n=0)
Lost to follow-up (n=3, in post-treatment year)
Discontinued intervention (n=0)
Analysis ‡
Analyzed (n=14)
Excluded from analysis (n=0)
Analyzed (n=14)
Excluded from analysis (n=0)
GDF15 assessment
GDF15 assessment
N=17
No spare serum available (n=0)
N=12
No spare serum available (n=2)
N=17
No spare serum available (n=0)
N=12
No spare serum available (n=2)
* Study started in January 2013 and completed in May 2016
§ Study started in December 2015 and completed in October 2019
‡ Patients who finalized the treatment and post-treatment phase with complete longitudinal data
